# Neural Transplants From Human Induced Pluripotent Stem Cells Rescue the Pathology and Behavioral Defects in a Rodent Model of Huntington’s Disease

**DOI:** 10.3389/fnins.2020.558204

**Published:** 2020-09-18

**Authors:** Yongwoo Yoon, Hyun Sook Kim, Chang Pyo Hong, Endan Li, Iksoo Jeon, Hyun Jung Park, Nayeon Lee, Zhong Pei, Jihwan Song

**Affiliations:** ^1^Department of Biomedical Science, CHA Stem Cell Institute, CHA University, Seongnam-si, South Korea; ^2^Department of Neurology, CHA Bundang Medical Center, CHA University, Seongnam-si, South Korea; ^3^Theragen Etex Bio Institute, Suwon-si, South Korea; ^4^Department of Neurology, The First Affiliated Hospital of Sun Yat-sen University, Guangzhou, China; ^5^iPS Bio, Inc., Seongnam-si, South Korea

**Keywords:** Huntington’s disease, induced pluripotent stem cells, neural precursor cells, cell transplantation, behavioral recovery

## Abstract

Huntington’s disease (HD) is a devastating, autosomal-dominant inheritance disorder with the progressive loss of medium spiny neurons (MSNs) and corticostriatal connections in the brain. Cell replacement therapy has been proposed as a potential therapeutic strategy to treat HD. Among various types of stem cells, human-induced pluripotent stem cells (iPSCs) have received special attention to develop disease modeling and cell therapy for HD. In the present study, the therapeutic effects of neural precursor cells (NPCs) derived from a human iPSC line (1231A3-NPCs) were investigated in the quinolinic acid (QA)-lesioned rat model of HD. 1231A3-NPCs were transplanted into the ipsilateral striatum 1 week after QA lesioning, and the transplanted animals showed significant behavioral improvements for up to 12 weeks based on the staircase, rotarod, stepping, apomorphine-induced rotation, and cylinder tests. Transplanted 1231A3-NPCs also partially replaced the lost neurons, enhanced endogenous neurogenesis, reduced inflammatory responses, and reconstituted the damaged neuronal connections. Taken together, these results strongly indicate that NPCs derived from iPSCs can potentially be useful to treat HD in the future.

## Introduction

Huntington’s disease (HD) is an autosomal-dominant neurodegenerative disorder caused by the abnormal expansion of cytosine-adenine-guanine (CAG) repeats in exon 1 of the *huntingtin* gene, which encodes a 350-kDa protein termed Huntingtin (MacDonald, 1993). The disease onset typically occurs in middle-aged people in correlation with the length of the CAG expansion (Duyao et al., 1993; Aziz et al., 2018), and the mutation largely causes degeneration of striatal medium spiny neurons (MSNs), resulting in the atrophy of caudate nucleus and putamen, as well as disturbed functions of the basal ganglia in the patient’s brain. Typically, HD patients exhibit progressive impairment of cognitive, motor, and psychiatric functions (Landles and Bates, 2004). Currently, no proven therapy for HD which can mitigate its devastating clinical course is available.

Stem cell therapy has been proposed to restore the degenerated MSNs and reestablish the degenerating striatopallidal circuit (Bjorklund and Lindvall, 2000). In addition, stem cells can provide immune modulatory factors (Vazey et al., 2006; Connor, 2018). Clinical trials have been performed using human fetal neural progenitor cells over the past two decades; however, the results varied and the clinical benefits were not significant (Freeman et al., 2000; Bachoud-Levi et al., 2006). Furthermore, standardization and ethical issues associated with the use of aborted human fetal tissues caused serious limitations (Bjorklund, 1993; Vazey et al., 2006). To overcome these limitations, induced pluripotent stem cells (iPSCs) have emerged as useful candidate cells to treat HD. In HD, human iPSCs and their neural progenitors have been utilized to delineate the effects of the HD mutation and pathophysiological process and as a monitoring platform for new drug development. However, because HD is a genetic disease, correction of the mutated gene will be essential for autologous cell therapy (An et al., 2012; Csobonyeiova et al., 2020).

In the present study, the therapeutic potential of neural precursor cells (NPCs) derived from 1231A3 iPSCs (Nakagawa et al., 2014) in the quinolinic acid (QA)-lesioned rat model of HD was investigated. Intrastriatal injection of QA leads to the induction of cell death of MSNs with relative striatal interneurons (Ramaswamy et al., 2007). The QA-lesioned rat model mimics the pathology of HD patients and shows defects in motor functions, sensorimotor responses, and cognitive deficits (Klein et al., 2013). The restorative capacity of transplanted 1231A3-NPCs for behavioral and pathological features in the QA-lesioned rat HD model was evaluated using multiple behavioral tests, immunohistochemical (IHC) staining of cell survival and differentiation of the transplants, level of scar formation, and endogenous neurogenesis. The connection to the host cells was demonstrated with retrograde axonal tracing using Fluoro-Gold (FG; Molecular Probes, Eugene, OR, United States).

## Materials and Methods

### Ethics Statement

The present study was performed in accordance with the CHA University Institutional Animal Care and Use Committee on animal experiments (IACUC, approval no: 140013). The study followed the ARRIVE guidelines for the reporting of animal research (Kilkenny et al., 2010). Animals were housed in standard laboratory cages (12-h light/dark cycles, temperature 23 ± 1°C), two rats in each cage, with access to food and water *ad libitum*.

### Preparation of Neural Precursor Cells Derived From Induced Pluripotent Stem Cells (1231A3-NPCs)

The 1231A3 human iPSC line, originally generated from peripheral blood mononuclear cells (PBMCs) using episomal vectors (Nakagawa et al., 2014), was provided by the Center for iPS cell Research and Application (CiRA) at Kyoto University, Japan (Umekage et al., 2019). 1231A3 cells were maintained under feeder-free and xeno-free conditions using StemFit^TM^ (Ajinomoto, Tokyo, Japan) medium and cultured on 35-mm tissue culture dishes (BD, Franklin Lakes, NJ, United States; 353002) coated with 0.5 mg/cm^2^ iMatrix-511 (Matrixome, Osaka, Japan).

1231A3 cells were differentiated into NPCs in the initial differentiation medium consisting of DMEM/F12, 5% knockout serum replacement (KOSR), 10 μM SB-431542 (21-A94; Reagents Direct, Encinitas, CA, United States), and 100 nM LDN-193189 (36-F52; Reagents Direct) for 3 days. Afterward, the cells were changed to the neural induction medium (NIM) consisting of DMEM/F12, N2 supplement (17502-048; Gibco, Dublin, Ireland), 0.2 mM ascorbic acid (A4544; Sigma-Aldrich, St. Louis, MO, United States), 2 mM L-glutamine (LS002-01; Welgene, Seoul, South Korea), 3 mM D-glucose (G7021; Sigma-Aldrich), 1 mM sodium pyruvate (11360-070; Gibco), and 3 μM CHIR 99021 (4423; TOCRIS, Bristol, United Kingdom). The medium was changed every day for 2 days. When rosette-like structures were formed, the cells were plated on 20 μg/ml poly-L-ornithine (PLO) and 5 μg/ml laminin (LN)-coated tissue culture dishes in N2/B27 medium including NIM with 1 × B27 supplement (17504-044; Gibco) and 20 ng/ml bFGF (100-18B; PeproTech, Cranbury, NJ, United States) (Liu et al., 2006; Sugai et al., 2016). NPCs were maintained under the same conditions until transplantation (TP). NPCs were dissociated using TrypLE select (12563-011; Gibco) diluted 0.5 × in 0.5 mM EDTA (15575-020; Gibco).

### Generation of Rat Huntington’s Disease Models

The QA-lesioned rat HD model was established based on a previously described method (Jeon et al., 2012). Twenty-eight adult male Sprague-Dawley rats (Orient Bio, Seongnam, South Korea), weighing 220–250 g, were randomly divided into three experimental groups: (1) 1231A3-NPC TP group (*n* = 10, rats received QA injection and 1231A3-NPC TP, QA + 1231A3-NPC), (2) fibroblast TP group (*n* = 9, rats received QA injection and fibroblast TP, QA + fibroblast), and (3) sham group (*n* = 9, rats received QA injection and media injection, QA + media). For QA lesioning, rats were anesthetized with an intraperitoneally administered solution of 1% ketamine (30 mg/kg) and xylazine hydrochloride (4 mg/kg), and then positioned in a stereotaxic apparatus. Stereotaxic intrastriatal injection of QA (2,3-pyridinedicarboxylic acid, Sigma-Aldrich) was used to create unilateral lesions of the right striatum in all rats. Each rat was injected with 120 nmol of QA dissolved in 2 μl of PBS using 26G Hamilton syringe at the following locations: AP = +1.0 mm, ML = −2.5 mm, DV = −5.0 mm, and tooth bar = −2.5 mm from the bregma. The QA was injected over a period of 6 min, and the needle was left in place for an additional 5 min and slowly removed over 6 min.

During the procedure, rats were placed on a heating pad to maintain the body temperature at 37 ± 1°C. After surgical procedures, the animal and the surgical wound were monitored and evaluated at least once a day for 1 week.

### Intracerebral Transplantation of 1231A3-NPCs

Seven days after QA lesioning, the unilateral TP of 1231A3-NPCs into the ipsilateral striatum of the QA-lesioned site was performed under the same anesthesia conditions used for QA lesioning. Then, 2 μl of 1231A3-NPCs (200,000 cells/2 μl) were injected at AP = +1.5 mm, ML = −3.0 mm, DV = −5.0 mm, and tooth bar = −2.4 mm from the bregma. Next, 2 μl of human foreskin fibroblasts [BJ-1, 200,000 cells, American Type Culture Collection (ATCC), Manassas, VA, United States; CRL-2522] was injected in the fibroblast group, and 2 μl of suspension media was injected in the sham group. Animals were intraperitoneally injected with cyclosporine A (20 mg/kg; CKD Pharmaceuticals, Seoul, South Korea) 2 days before TP and then every day after TP (10 mg/kg).

### Behavioral Tests

Behavioral tests were conducted to determine the pathology of QA-induced striatal lesions and the restorative effects of transplanted 1231A3-NPCs on motor functions. Five tests were administered: the staircase test, rotarod test, stepping test, apomorphine-induced rotation test, and cylinder test. Prior to the behavior tests, rats were trained under the same conditions three times per day for three consecutive days to reduce variation in each animal. All animals were tested at 8 time points; 1 day before QA injection (pre), 6 days after QA lesioning (baseline, 0 week), and every 2 weeks post-TP in a 12-week period (post-TP 2, 4, 6, 8, 10, and 12 weeks). Cylinder test was performed only once at 12 weeks post-TP (Supplementary Figure S1). In the staircase test, food pellets were placed on the seven staircases with five food pellets placed on each step. Water and food supply was restricted for 3 days prior to and during the training or test. Then, all animals were allowed to retrieve pellets from each step on the left side of the staircase box. For 15 min, the total number of pellets retrieved by each animal was recorded (Montoya et al., 1991). In the rotarod test, the average time to stay on the rotarod from three trials was recorded for all rats on the acceleration mode of progressively increasing rod speeds from 4 to 40 rpm for 5 min (Song et al., 2006; Hanna et al., 2007). In the stepping test, the rats were slowly moved sideways placing their forepaws along the 900-mm-wide band on the table for 5 s and the number of forepaw placings was counted (Olsson et al., 1995; Kirik et al., 1998). For the apomorphine-induced rotation test, animals were injected with apomorphine (1.0 mg/kg in normal saline containing 0.2% ascorbate; Sigma-Aldrich) and placed in an acrylic box cage individually for a 5-min habituation period before a 60-min test session. The number of complete 360° ipsilateral turns (rotation) and the difference between the ipsilateral and the contralateral turns were counted (Nakao and Itakura, 2000). In the cylinder test, animals were placed in a transparent Plexiglas cylinder and the number of independent wall placements observed for the right forelimb, left forelimb, and both forelimbs simultaneously, was recorded three times. An asymmetry in forelimb use during vertical exploration was calculated once at 12 weeks post-TP (Hua et al., 2002).

### Histological Analysis

At 13 weeks post-TP, all animals (*n* = 28) were anesthetized by intraperitoneal injection of 1% ketamine (30 mg/kg) and xylazine hydrochloride (4 mg/kg), and then perfused transcardially with heparinized (5 U/ml) saline and 4% buffered paraformaldehyde. The rat brains were sequentially prepared for histological analysis using overnight fixation in 4% paraformaldehyde at 4°C, 30% sucrose solution for 2 days until they sank, and stored at −80°C in Tissue-Tek O.C.T compound (Leica, Lot No. 3801480). After fixation, 40-μm-thick frozen coronal sections were prepared using a cryostat (Leica CM3050 S; Leica Microsystems, Germany) for immunohistochemistry.

### Fluoro-Gold Injection

To determine whether transplanted cells were functionally connected to the host tissue, the retrograde tracer, FG, was injected intracerebrally into the ipsilateral external segment of the globus pallidus (GP) of the QA-lesioned site under the same anesthesia conditions used for QA lesioning. Two randomly selected rats from each group were used. At 12 weeks post-TP, 0.5 μl of 4% FG was injected using a 26G Hamilton syringe into the following sites: AP = −1.0 mm, ML = −2.5 mm, DV = −6.0 mm, and tooth bar = −2.5 mm from the bregma. Rats were killed 1 week after the FG injection (i.e., 13 weeks post-TP).

### 5-Bromo-2′-Deoxyuridine Labeling

To determine the level of cell proliferation in the subventricular zone (SVZ) in the QA-lesioned brain, the thymidine analog, 5-bromo-2′-deoxyuridine (BrdU, Sigma-Aldrich), was used to label proliferating (S-phase) cells (Miller and Nowakowski, 1988). Rats were injected with 300 μl of BrdU (50 mg/kg) intraperitoneally two times at 12-h intervals every day for 3 days before perfusion. Three randomly selected rats from each group were used.

### Immunohistochemistry

Three independent sections from each animal in every experimental group were used for IHC analysis for each marker. To detect transplanted human cells and MSNs, 3,3′-diaminobenzidine (DAB) immunohistochemistry was performed on free-floating sections of brain tissues using primary antibodies against human-specific nuclei (hNu) and dopamine- and cAMP-regulated phosphoprotein Mr 32 kDa (DARPP-32), respectively. In parallel, double-label immunofluorescence was performed on free-floating sections as well. In either case, free-floating sections were washed two times for 15 min with PBS, three times for 10 min with PBS/0.3% Triton X-100 (Sigma-Aldrich), and then blocked for 60 min using 5% normal horse serum (Vector Laboratories, Burlingame, CA, United States) in PBS/0.3% Triton X-100 at room temperature. For BrdU immunohistochemistry, tissue sections were pretreated in 0.1 M sodium citrate buffer for 30 min at 100°C before incubation with the antibody (Tang et al., 2007).

Afterward, sections were incubated at 4°C for 14 h with primary antibodies against the following: hNu (mouse anti-human nuclei, 1:200, Millipore, Billerica, MA, United States), human-specific mitochondria (hMito) (rabbit anti-mitochondria, 1:100, Millipore), medium spiny neurons (DARPP-32) (rabbit anti-darpp32, 1:250, Cell Signaling, Danvers, MA, United States), microtubule-associated protein (MAP2) (rabbit anti-microtubule-associated protein 2, 1:250, Millipore), γ-aminobutyric acid (GABA) (rabbit anti-GABA, 1:200, Sigma-Aldrich), both molecular forms of glutamic acid decarboxylase (GAD65/67) (rabbit anti-glutamate decarboxylase 65/67, 1:250, Millipore), NPC marker (hNestin) (rabbit anti-nestin, 1:200, Covance, Princeton, NJ, United States), oligodendrocyte lineage (O4) (mouse anti-O4, 1:100, Millipore), synaptophysin (SVP-38) (mouse anti-synaptophysin, 1:200, Sigma-Aldrich), BrdU (mouse anti-BrdU, 1:200, BD Biosciences, Franklin Lakes, NJ, United States), doublecortin (DCX) (rabbit anti-doublecortin, 1:200, Cell Signaling), activated microglia (ED1) (mouse anti-CD68, 1:100, Serotec), glial fibrillary acidic protein (GFAP) (mouse anti-GFAP, 1:250, BD Biosciences), macrophage mannose receptor (CD206) (goat anti-CD206, 1:200, Santa Cruz Biotechnology, Dallas, TX, United States), NOS2 (iNOS) (rabbit anti-NOS2, 1:200, Santa Cruz Biotechnology), and FG (rabbit anti-Fluoro-Gold, 1:1,000, Fluorochrome LCC, Denver, CO, United States).

For double-label immunofluorescence staining, sections were washed five times for 10 min with PBS and incubated with appropriate fluorescence-conjugated secondary antibodies against the primary antibodies for 90 min. Secondary goat anti-mouse IgG-conjugated Alexa 488 and 555 and goat anti-mouse IgM-conjugated Alexa 555 were used for immunofluorescence labeling (1:500, Molecular Probes). Sections were subsequently washed for 10 min with PBS and then incubated in 4′,6-diamidine-2-phenylindole dihydrochloride (DAPI, 1:500, Roche, Indianapolis, IN, United States) for 15 min to counterstain the cell nuclei. Fluorescence-labeled images were captured using a confocal laser-scanning microscope (LSM510, Carl Zeiss Microimaging, Inc., Jena, Germany). Histological changes were quantified using ImageJ (NIH). Counting for each marker expression was performed manually (i.e., DARPP-32, GABA, and GAD65/67 for striatal neuron differentiation; DCX and BrdU for neurogenesis; ED1, iNOS, and CD206 for macrophage responses; GFAP for glial scar formation) in three separate 100 μm^2^ regions of interest (ROI) from each of three sections per rat.

### Statistical Analysis

Statistical analyses were performed using the statistical analysis system (SAS version 8, SAS Institute, Inc., Cary, NC, United States). Statistical analyses of the behavior and imaging data of the number of cells positively stained with DARPP-32, GABA, GAD65/67, BrdU, DCX, ED1, GFAP, CD206, and iNOS were performed. Repeated measurements of behavioral data were analyzed using PROC MIXED procedure of a linear mixed model procedure. The differences in the mean values among the TP groups were greater than would be expected by chance (*p* < 0.001). For imaging data analysis, the one-way analysis of variance with Fisher’s least significant difference method was performed. Shapiro–Wilk test was applied for testing the normality of data. The results were presented as the mean ± standard error of the mean (SEM). A *p*-value < 0.05 was considered statistically significant.

## Results

### Behavioral Improvements Following Transplantation of 1231A3-NPCs Into the Quinolinic Acid-Lesioned Rat Model

**FIGURE 1:**
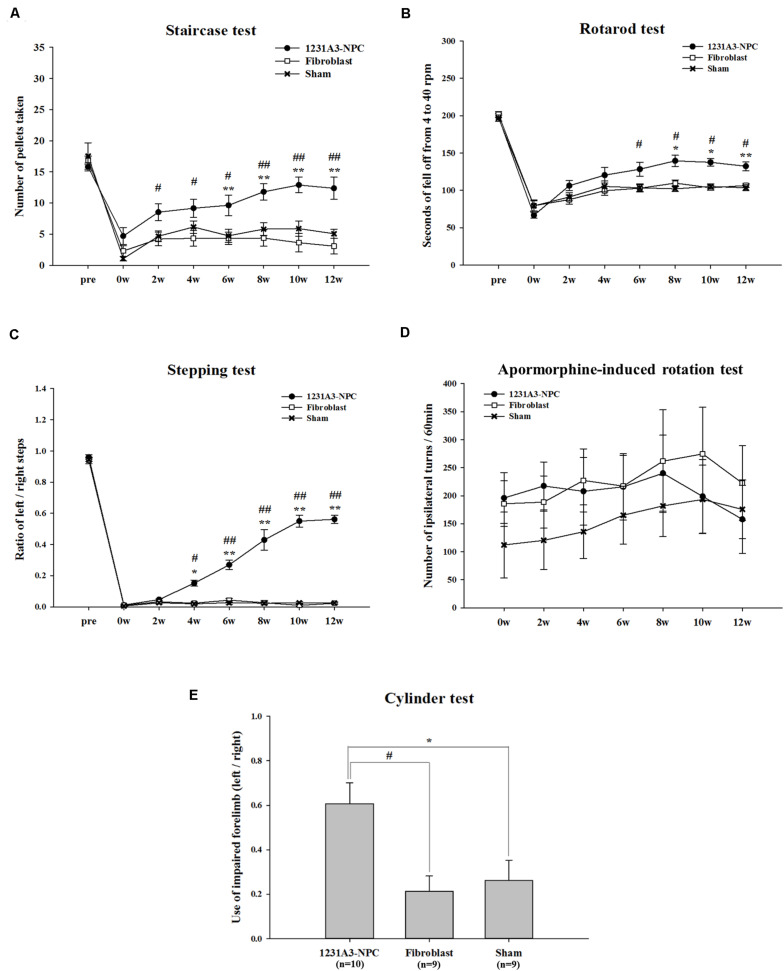
Results of behavioral assessment in the 1231A3-NPC TP, fibroblast TP, and sham groups after TP into the QA-lesioned rat model. **(A)** Staircase test. **(B)** Rotarod test. **(C)** Stepping test. **(D)** Apomorphine-induced rotation test. **(E)** Cylinder test. *n* = 10, 9, and 9 in the 1231A3-NPC TP group, fibroblast TP group, and sham group, respectively. [1231A3-NPC TP vs. fibroblast TP (^#^*p* < 0.05; ^##^*p* < 0.001), 1231A3-NPC TP vs. sham (*p < 0.05; **p < 0.001)]. NPCs, neural precursor cells; TP, transplantation; QA, quinolinic acid.

To investigate whether 1231A3-NPCs could reduce behavioral impairment in the QA-lesioned rat HD model, five behavioral tests were performed (staircase, rotarod, stepping, apomorphine-induced rotation, and cylinder) up to 12 weeks following TP. The 1231A3-NPC TP group (*n* = 10) showed significant behavioral improvements compared with the fibroblast TP (*n* = 9) and sham (*n* = 9) groups (Figure 1).

In the staircase test, the 1231A3-NPC TP group showed a gradually increased number of retrieved pellets compared with the fibroblast TP group (*p* < 0.04 for the 1231A3-NPC TP group compared with the fibroblast TP group) until 6 weeks post-TP. The differences in the number of retrieved pellets were greater in the 1231A3-NPC TP group compared with the fibroblast TP and sham groups from 8 weeks post-TP (*p* < 0.0001 for the 1231A3-NPC TP group compared with the fibroblast TP group, *p* < 0.04 for the 1231A3-NPC TP group compared with the sham group; Figure 1A).

In the rotarod test, the mice in the 1231A3-NPC TP group fell off significantly later compared with mice in the fibroblast TP group from 6 weeks post-TP and the sham group from 8 weeks post-TP. Group difference of falling latency increased progressively over time (*p* < 0.03 for the 1231A3-NPC TP group compared with the fibroblast TP group at 6 weeks post-TP, *p* < 0.01 for the 1231A3-NPC TP group compared with the sham group at 8 weeks post-TP, and *p* < 0.001 for the 1231A3-NPC TP group compared with the sham group at 12 weeks post-TP; Figure 1B).

In the stepping test, 1231A3-NPC TP group exhibited a significant behavioral improvement compared with the fibroblast TP and sham groups (*p* < 0.01 for the 1231A3-NPC TP group compared with the fibroblast TP and sham groups at 4 weeks post-TP and *p* < 0.0001 for the 1231A3-NPC TP group compared with the fibroblast TP and sham groups at 6 weeks post-TP; Figure 1C).

In the apomorphine-induced rotation test, the number of ipsilateral turns returned to the baseline measurements from 10 to 12 weeks post-TP in the 1231A3-NPC TP group. In contrast, the level of ipsilateral rotational asymmetry was progressively increased in the sham group compared with baseline, and the fibroblast TP group showed no difference from the baseline measurements 12 weeks post-TP (Figure 1D). This finding indicates that QA lesioning induced rotational asymmetry could be normalized after 1231A3-NPC TP; however, the asymmetry deteriorated progressively as shown in the sham group.

In the cylinder test, locomotor asymmetry of QA-lesioned rats was evaluated once at 12 weeks post-TP, and the ratio of intact forelimb to the impaired forelimb use was 0.607 ± 0.093 in the 1231A3-NPC TP group, 0.212 ± 0.070 in the fibroblast TP group, and 0.261 ± 0.091 in the sham group. The use of impaired forelimb was significantly increased in the 1231A3-NPC TP group compared with the fibroblast TP and sham groups (*p* < 0.01 for the 1231A3-NPC TP group compared with the fibroblast TP and sham groups, Figure 1E).

Recovery rate was calculated from staircase, rotarod, and stepping tests, and the 1231A3-NPC TP group showed 69.1 ± 16.0, 50.0 ± 4.5, and 58.0 ± 2.8% recovery rate from baseline, respectively. However, recovery rate in the staircase, rotarod, and stepping tests was 5.3 ± 9.1, 22.1 ± 6.2, and 1.3 ± 0.7% in the fibroblast TP and 24.3 ± 4.5, 20.6 ± 3.4, and 2.0 ± 0.7% sham groups, respectively (Table 1).

**TABLE 1:** Behavioral recovery rates in the 1231A3-NPC TP, fibroblast TP, and sham groups (mean ± SEM %).

Group	1231A3-NPCs (*n* = 10)	Fibroblast (*n* = 9)	Sham (*n* = 9)
**Recovery rate (%)**			
Staircase test	69.1 ± 16.0	5.3 ± 9.1	24.3 ± 4.5
Rotarod test	50.0 ± 4.5	22.1 ± 6.2	20.6 ± 3.4
Stepping test	58.0 ± 2.8	1.3 ± 0.7	2.0 ± 0.7

*Recovery rate = measurements at 12 weeks post-TP/measurements before QA lesioning. *NPC*, neural precursor cell; *TP*, transplantation; *SEM*, standard error of the mean; *QA*, quinolinic acid.*

In conclusion, these results strongly indicated that TP of 1231A3-NPCs can improve the behavioral defects in the QA-lesioned HD rat model.

### Distribution of 1231A3-NPCs and Restoration of DARPP-32-Positive Cells Following Transplantation Into Quinolinic Acid-Lesioned Rat Models

**FIGURE 2:**
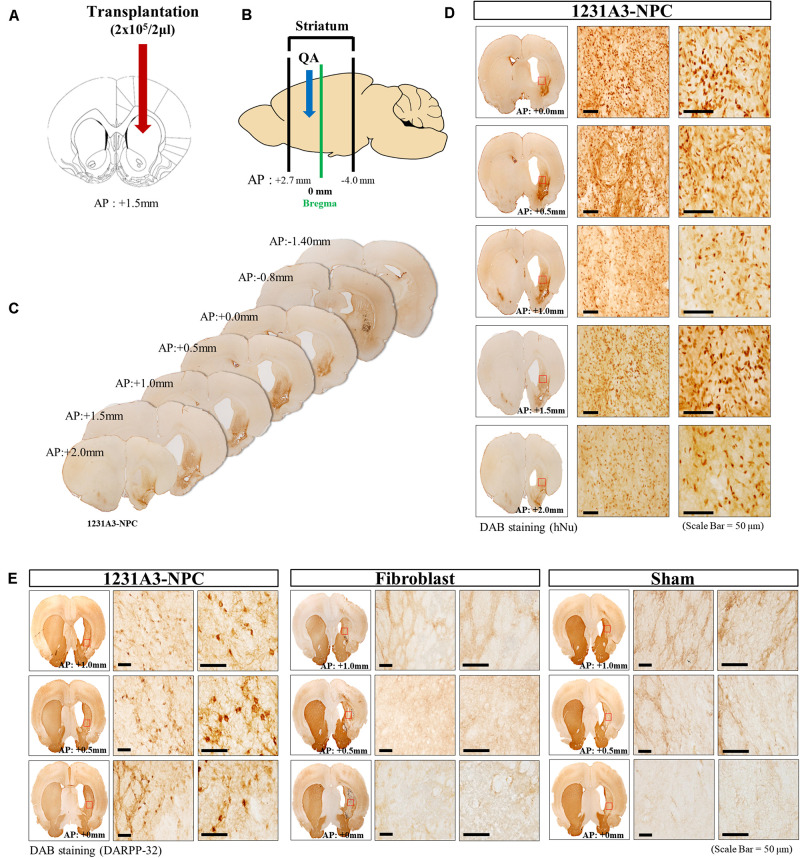
Representative distribution patterns of transplanted 1231A3-NPCs and restoration of DARPP-32-positive cells in the QA-lesioned site. **(A,B)** Location of 1231A3-NPC injection and QA-lesioned striatum. **(C)** Serial brain sections of mice in the 1231A3-NPC TP group. **(D)** DAB IHC staining using an antibody against hNu in the QA-lesioned striatum. **(E)** DAB staining using an antibody against DARPP-32 in the QA-lesioned striatum. Scale bar = 50 μm. NPC, neural precursor cell; QA, quinolinic acid; TP, transplantation; DAB, 3,3′-diaminobenzidine; IHC, immunohistochemical; hNu, human-specific nuclei; DARPP-32, dopamine- and cAMP-regulated neuronal phosphoprotein.

Cell suspension (2 μl) was injected into the QA-induced striatal lesion (Figure 2A), which ranged from AP +2.7 to -4.0 mm (Figure 2B). The distribution of transplanted 1231A3-NPCs was identified using DAB IHC staining with an antibody against hNu; the 1231A3-NPCs were mainly distributed from AP +2.0 to 0.0 mm on the QA-induced, clear striatal lesion (Figures 2C,D). To confirm the restoration of striatal MSNs, DAB IHC staining using an antibody against DARPP-32 was performed; the DARPP-32-positive cells were found only in the 1231A3-NPC TP group compared with the fibroblast TP and sham groups (Figure 2E). These findings demonstrated that 1231A3-NPCs could survive up to 13 weeks post-TP and restore medium-sized striatal projection neurons expressing DARPP-32 in the QA-lesioned site.

### Neuronal Differentiation of Transplanted 1231A3-NPCs in the Quinolinic Acid-Lesioned Site

**FIGURE 3:**
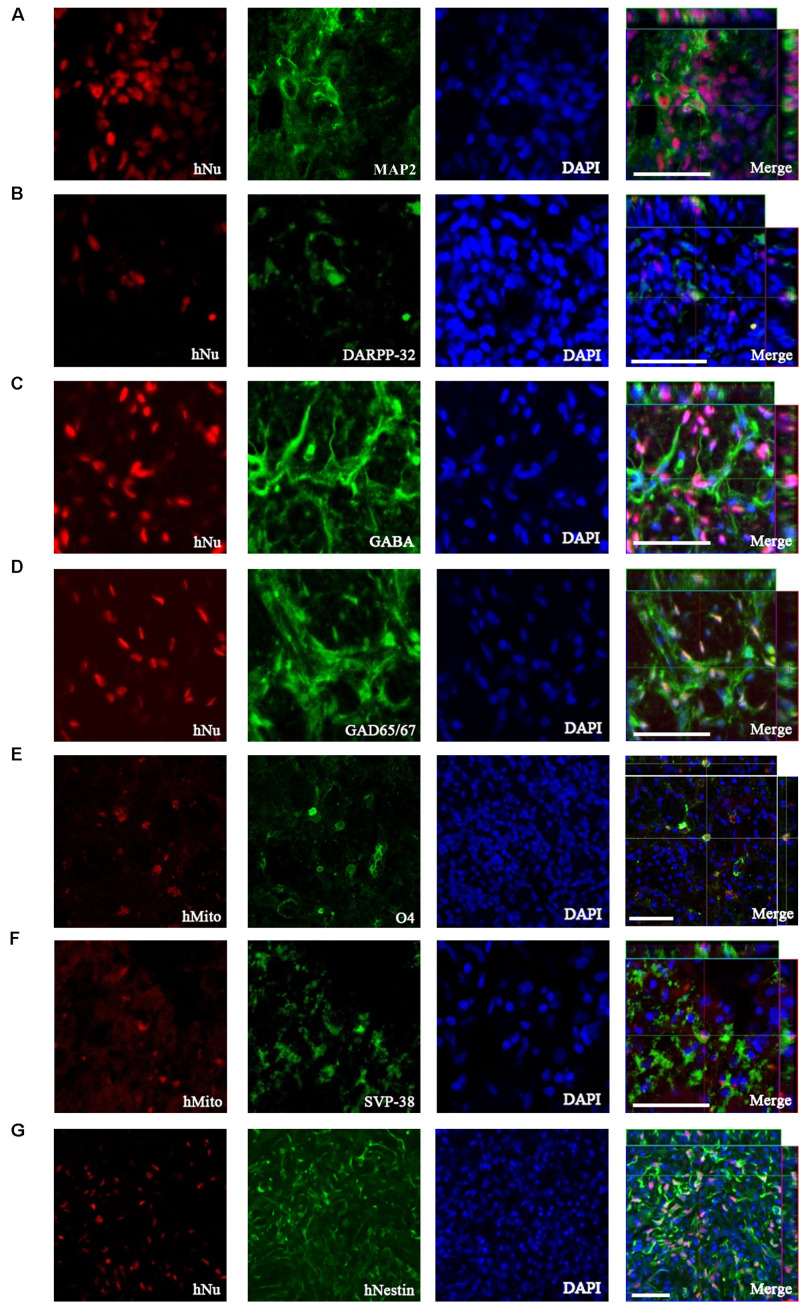
IHC analysis showing the neuronal differentiation caused by transplanted 1231A3-NPCs. Antibodies against either hNu or hMito show the transplanted 1231A3-NPCs were co-localized with **(A)** MAP2, **(B)** DARPP-32, **(C)** GABA, **(D)** GAD65/67, **(E)** O4, **(F)** SVP-38, and **(G)** hNestin antigens. Scale bar = 50 μm. IHC, immunohistochemical; NPCs, neural precursor cells; DAPI, 4′,6-diamidine-2-phenylindole dihydrochloride; GABA, gamma-aminobutyric acid; GAD, glutamic acid decarboxylase; hMito, human-specific mitochondria; hNu, human-specific nuclei antigen; MAP2, microtubule-associated protein; SVP-38, synaptophysin.

To investigate neuronal differentiation following TP of 1231A3-NPCs into the QA-lesioned site showing neuronal loss and striatal atrophy, IHC analysis was performed using antibodies against hNu and hMito. At 13 weeks post-TP, 1231A3-NPCs were differentiated into MAP2-positive mature neurons (Figure 3A), including DARPP-32-positive MSNs and GABA- and GAD65/67-positive GABAergic neurons (Figures 3B–D). The efficiency rate of neuronal differentiation for DARPP-32, GABA, and GAD65/67 was 4.4 ± 0.78%, 7.9 ± 1.2%, and 6.5 ± 1.1%, respectively. In addition, 1231A3-NPCs had differentiated into O4-positive oligodendrocytes and expressed a synaptic vesicle protein, synaptophysin (SVP-38) (Figures 3E,F). Confocal analysis indicated that some surviving 1231A3-NPCs remained as hNestin-positive neural precursors (Figure 3G).

### Increase in Endogenous Neurogenesis Caused by Transplanted 1231A3-NPCs

**FIGURE 4:**
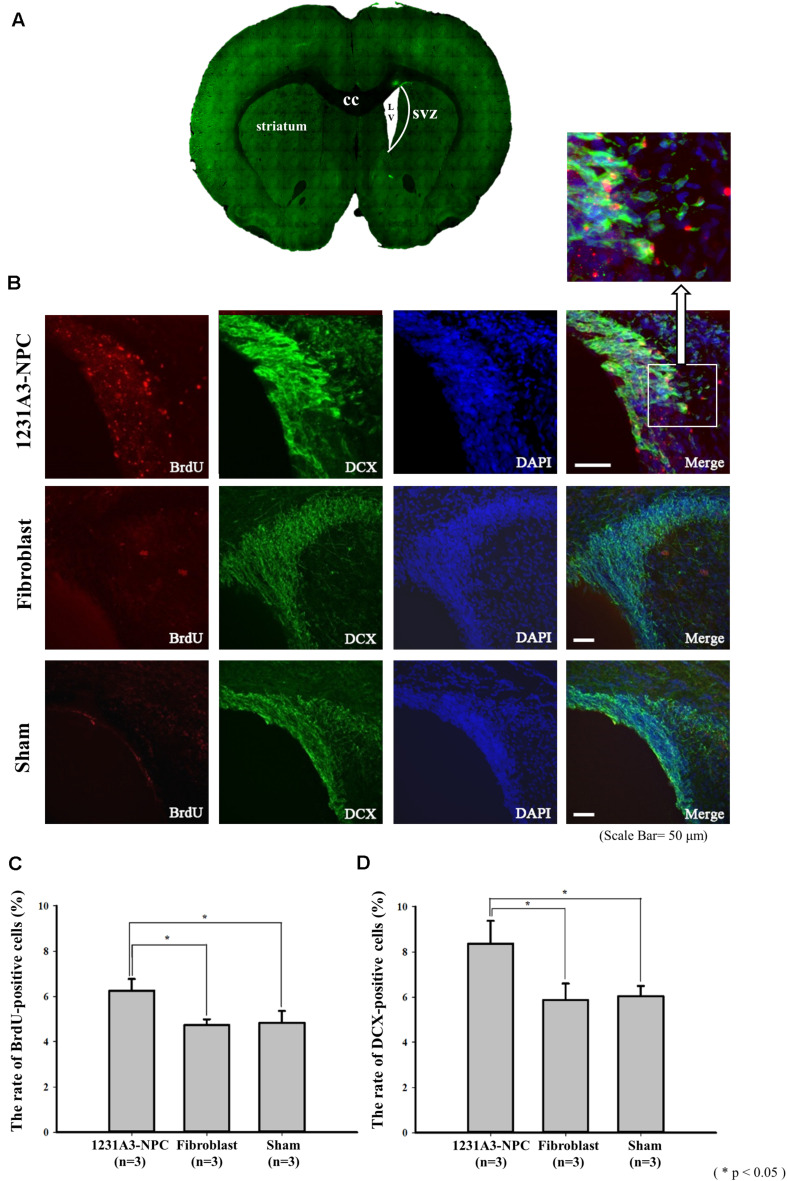
IHC analysis showing the increase of endogenous neurogenesis by transplanted 1232A3-NPCs. **(A)** Coronal section of ipsilateral QA-lesioned rat model. **(B)** BrdU- and DCX-positive cells in ipsilateral SVZ 13 weeks post-TP. Scale bar = 50 μm. Arrow indicates the enlarged image in the inset. **(C,D)** Bar graph showing the percentage of BrdU-positive cells and the percentage of DCX-positive cells (*n* = 3 in each group). IHC, immunohistochemical; NPCs, neural precursor cells; QA, quinolinic acid; TP, transplantation BrdU, 5-bromo-2′-deoxyuridine; cc, corpus callosum; LV, lateral ventricle; SVZ, subventricular zone; *p < 0.05.

To determine whether 1231A3-NPCs could increase endogenous cell proliferation in the QA-lesioned SVZ (Figure 4A), double immunostaining was performed using BrdU and SVZ neuroblast marker (DCX) (Figure 4B). The number of BrdU-positive cells and DCX-positive neuroblasts in the ipsilateral SVZ was significantly increased in the 1231A3-NPC TP group compared with fibroblast TP and sham groups 13 weeks post-TP (*p* < 0.05 for the 1231A3-NPC TP group compared with the fibroblast TP group, *p* < 0.05 for the 1231A3-NPC TP group compared with the sham group, Figures 4C,D). In the 1231A3-NPC TP group, the BrdU/DCX double-labeled cells were also found at the margin of SVZ toward the damaged striatal lesion (enlarged view in Figure 4B). These findings indicate that 1231A3-NPC TP could enhance proliferation and migration of neuroblasts in the SVZ after QA lesioning, which might lead to the formation of new neurons in the striatum.

### Reduced Glial Scar Formation and Inflammatory Responses Caused by Transplanted 1231A3-NPCs

**FIGURE 5:**
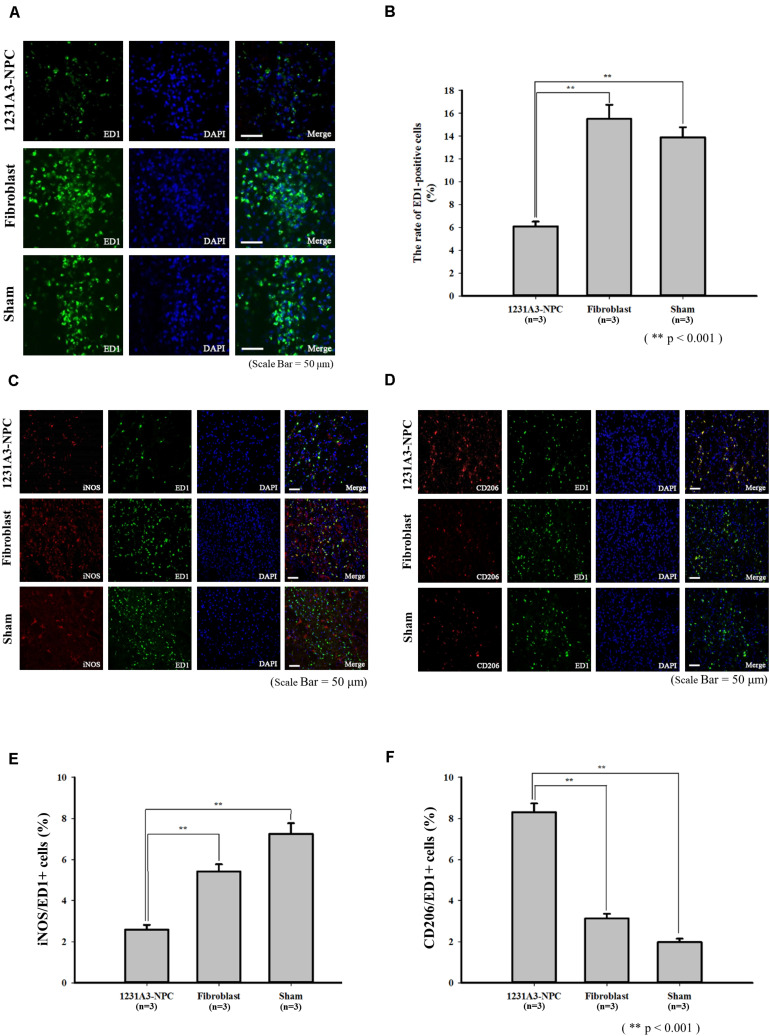
Effects of 1231A3-NPCs on inflammatory responses in QA-lesioned rat model 13 weeks post-TP. **(A)** Activated microglia marker (ED1) staining. **(B)** Bar graph showing the number of ED1-positive cells (*n* = 3 in each group). **(C)** Proinflammatory cytokine (iNOS) staining. **(D)** Anti-inflammatory cytokine (CD206) staining. **(E,F)** Bar graph showing the rate of iNOS-positive cells and CD206-positive cells (*n* = 3 in each group). Scale bar = 50 μm, **p < 0.001. NPCs, neural precursor cells; QA, quinolinic acid; TP, transplantation.

To evaluate the effect of 1231A3-NPCs on glial scar formation in the QA-lesioned rat model, IHC analysis using an astrocyte marker, GFAP, was performed at 13 weeks post-TP. In addition, the changes in inflammatory responses caused by transplanted 1231A3-NPCs were examined using antibodies against activated microglial marker (ED1), M1 proinflammatory cytokine (iNOS), and M2 anti-inflammatory cytokine (CD206). The ratio of ED1-positive activated microglia was decreased in the 1231A3-NPC TP group compared with fibroblast TP and sham groups (Figure 5A). Decreased proinflammatory cytokine (iNOS) of M1 microglia and increased anti-inflammatory cytokine (CD206) of M2 microglia were found in the 1231A3-NPC TP group compared with the fibroblast TP and sham groups (Figures 5B–F). The ratio of GFAP-positive cells was also decreased in the 1231A3-NPC TP group compared with the fibroblast TP and sham groups (*p* < 0.001, Supplementary Figure S2).

### Neuronal Connection of Transplanted 1231A3-NPCs to the Host Tissue

**FIGURE 6:**
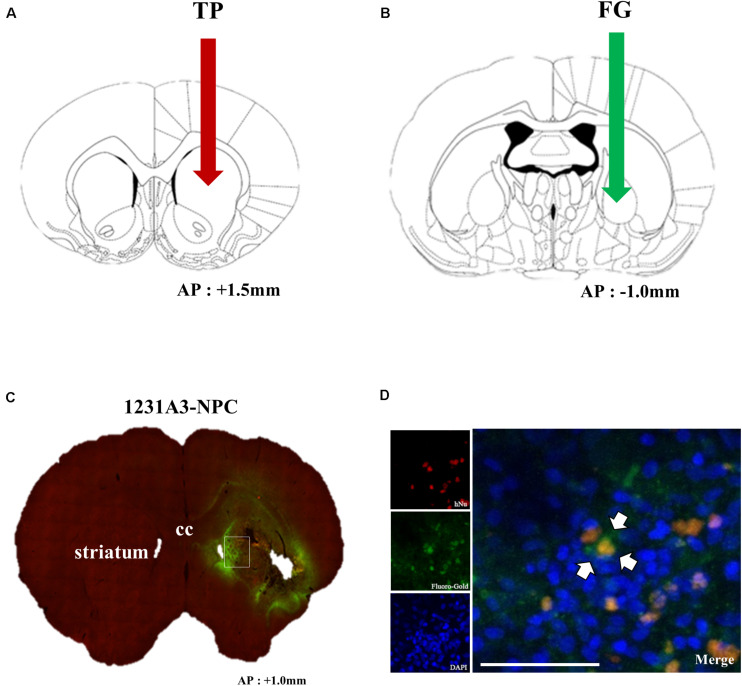
Assessment of the connection of transplanted 1231A3-NPCs to the host tissue using FG labeling. **(A)** Location for intrastriatal TP of 1231A3-NPCs. **(B)** Location of the FG injection 12 weeks post-TP. **(C)** Distribution of FG signals in the whole brain sections. **(D)** Confocal image of double immunostaining with antibodies against hNu and FG in the striatum. Scale bar = 50 μm. NPCs, neural precursor cells; FG, Fluoro-Gold; TP, transplantation; hNu, human-specific nuclei. Arrows in Figures **(A,B)** indicate TP sites.

To confirm whether intrastriatal TP of 1231A3-NPCs (Figure 6A) can be functionally connected to the host neural networks, a retrograde tracer, FG, was injected into the GP (Figure 6B) 1 week prior to killing, and double immunostaining was performed using hNu and FG. Notably, co-expressed hNu- and FG-positive cells were observed in the ipsilateral striatum near the TP site (Figure 6C). Co-localization of hNu- and FG-positive cells in the striatum (Figure 6D) indicates that GP-injected FG was taken up into the host cells and transported to the 1231A3-NPCs through synapses, demonstrating the formation of connections between the transplanted cells and the host tissue.

## Discussion

In the present study, the therapeutic effects of human iPSC-derived NPCs (1231A3-NPCs) following TP into a QA-lesioned HD rat model were investigated. *In vivo* effects of 1231A3-NPCs on behavioral changes were assessed using the staircase, rotarod, stepping, cylinder and apomorphine-induced rotation tests until 12 weeks post-TP. Behavioral performances before and after the 1231A3-NPC TP were analyzed in comparison with the fibroblast TP and sham groups. At 13 weeks post-TP, histological changes were assessed for survival, differentiation, neural connections of transplanted cells, and their effects on endogenous neurogenesis, as well as gliosis and inflammatory responses.

Behaviorally, the 1231A3-NPC TP group showed markedly improved motor deficits compared with the fibroblast TP and sham groups, and the recovery rates were over 50% compared with baseline in the staircase, rotarod, and stepping tests. Improvement of motor function was detected as early as 2 weeks post-TP in the skilled reaching, 4 weeks in forearm akinesia, and 8 weeks in general motor and balance. These differences in motor function continued to increase until 10 weeks post-TP and persisted until the end of behavioral evaluation. Because a certain amount of time for the transplanted cells to give rise to the replacement or restoration of lesioned striatum was anticipated, the initial improvement in the skilled reaching was likely due to paracrine effect rather than neuronal differentiation or neural connection to host cells. In our previous work, TP of HD72-iPSC-derived neural precursors were shown to restore behavioral deterioration as early as 3 weeks (Jeon et al., 2014). Therefore, 1231A3-NPCs, which are derived from a healthy donor, can likely produce a better result compared with neural cells differentiated from HD patient-derived iPSCs.

The IHC analyses showed the transplanted 1231A3-NPCs could survive up to at least 13 weeks post-TP, and the ratio of differentiation into mature striatal neurons ranged from 4.4 ± 0.78% for DARPP-32 to 7.9 ± 1.2% for GABA. Because neural stem cells (NSCs) show a poor survival rate and modest treatment effects *in vivo* (Tang et al., 2017), evaluating the survival and differentiation ratio after TP of NPCs or NSCs is important. In previous studies, approximately 5–70% of transplanted NPCs derived from iPSCs or hESCs differentiated into various types of mature neurons (Oki et al., 2012; Polentes et al., 2012; Tornero et al., 2013, 2017; Yuan et al., 2013; Hermanto et al., 2018). Therefore, the number of cells or any procedures to facilitate the neural differentiation for 1231A3-NPCs should be redefined for further clinical use.

As previously suggested, the QA-lesioned HD model showed an impaired endogenous neurogenesis in the SVZ, thus, 1231A3-NPCs were expected to recover the neurogenesis in the SVZ of the QA-lesioned site (Vazey et al., 2006; Hermann et al., 2014). In the present study, the number of BrdU-positive proliferating cells and DCX-positive neuroblasts were significantly increased in the ipsilateral SVZ, strongly indicating the endogenous neurogenesis was enhanced in the 1231A3-NPC TP group. Although no obvious fusiform-shaped cell bodies with single leading and/or trailing processes were observed from the SVZ to the lesioned striatum, indicative of the migration of newly generated neural cells (Tattersfield et al., 2004; Zheng et al., 2006), the increased DCX- and BrdU-positive cells located on the margin of SVZ toward the damaged striatal side (enlarged view in Figure 4B) likely possessed the potential to migrate to and repair the damaged striatum (Okano et al., 2007).

Expression of SVP-38 and the detection of FG in the striatum support the capacity of differentiated neurons from 1231A3-NPCs forming synaptic vesicles and being connected to the distant host neurons *in vivo*. These findings indicate the possibility of reconstituted synapse formation and neural connections between 1231A3-NPCs in the lesioned striatum and the host tissue in the GP where FG was injected. Retrograde neuronal tracing analysis using FG examines the capacity of neural connections; however, there are several alternative methods to investigate neural connections such as neuronal tracing using deletion-mutant rabies virus (Wickersham et al., 2007) or monitoring synaptic transmission using dual whole-cell patch-clamp recording in brain slices (Petersen, 2017). Because the neural connectivity and physiological function of differentiated neurons are essential features for clinical use, using multiple methods to demonstrate neural connections would be helpful.

In the QA-lesioned HD rat model, activation of the brain inflammatory process can mimic the extensive astrocytosis and microgliosis in the brains of HD patients (Moresco et al., 2008). The results of the present study showed TP of 1231A3-NPCs ameliorated the proinflammatory environment in the QA-lesioned striatum by reducing the expression of proinflammatory cytokine (iNOS) of M1 microglia and increasing anti-inflammatory cytokine (CD206) of M2 microglia. In addition, the decrease of ED1-positive activated microglia and GFAP-positive scar formation was observed.

Xenotransplantation of HD72-iPSC-derived neural precursors into the striatum of YAC128 mice improved behavioral deterioration, and GABAergic neuronal differentiation was observed (Jeon et al., 2014). Intrastriatal TP of allogeneic mouse iPSC-NSCs into YAC128 mice showed marked cell survival, region-specific neuronal differentiation (i.e., MSN), and no sign of tumors, as well as beneficial effects on behavioral deficits and neuropathological changes (Al-Gharaibeh et al., 2017). Behavioral and histological findings in the present study are compatible with the previous preclinical stem cell studies.

Safety issues may include TP-related infection, allograft-related rejection, and unexpected transplant behavior *in vivo*, leading to tumor formation (Herberts et al., 2011). Among the safety issues, tumor formation is the major concern when using pluripotent stem cells, especially when the transplanted cells remain undifferentiated *in vivo*. In previous studies, the majority of Nestin-positive, undifferentiated neural precursor cells, were non-proliferative at TP (Bernal and Arranz, 2018; Vonderwalde et al., 2020), which could be due to the NPC’s characteristics of retaining their original features and/or the microenvironmental interactions from the transplanted brain. In particular, TP of 1231A3 iPSC-induced neural stem/progenitor cells, in which the identical cell source with similar neural differentiation potential as used in this study, reportedly exhibited no evident carcinogenesis in immunodeficient mice (Sugai et al., 2016). Furthermore, the presence of hNestin-positive neural precursors at 13 weeks post-TP can be of significant concern because the remaining stemness of transplants can cause tumor formation. The potential risk of tumorigenicity cannot be fully excluded in the present study, although no indication of tumor formation was observed in the analysis. Therefore, long-term follow-up studies and various types of evaluations based on global consensus will be useful to address in the future the possibility of tumorigenicity of transplanted NPCs (Lee et al., 2013; Sato et al., 2019).

In summary, five unique features of the therapeutic effects caused by transplanted 1231A3-NPCs were observed in the present study: (1) Transplanted cells produced a rapid and significant behavioral improvement; (2) Transplanted cells restored DARPP-32-positive MSNs in the lesioned striatum; (3) Transplanted cells formed GABA- and MSN-type neurons, relevant for functional recovery; (4) Transplanted animals showed an increase of endogenous neurogenesis and decrease of inflammatory responses in the damaged host brain; and (5) Transplanted cells could reconstitute the damaged neuronal connections. These features of neural precursor cells derived from iPSCs were demonstrated in the treatment of a preclinical HD animal model, which will serve as an experimental basis for developing iPSC-based clinical trials for HD in the future.

## Data Availability Statement

The raw data supporting the conclusions of this article will be made available by the authors, without undue reservation.

## Ethics Statement

The animal study was reviewed and approved by the CHA University Institutional Animal Care and Use Committee on animal experiments (IACUC, approval no: 140013).

## Author Contributions

YY, HSK, and JS were responsible for the study concept and design. YY, EL, HJP, NL, and ZP were responsible for data acquisition. YY, HSK, CPH, and JS performed data analysis and manuscript writing. JS finalized the manuscript. All authors have read and approved the final version of the manuscript.

## Conflict of Interest

JS is the founder and CEO of iPS Bio, Inc. The remaining authors declare that the research was conducted in the absence of any commercial or financial relationships that could be construed as a potential conflict of interest. The authors declare that this study received funding from iPS Bio, Inc. The funder had the following involvement with the study: study design, data analysis and interpretation, and manuscript writing.

## Supplementary Material

FIGURE S1 Schematic representation of experimental designs. Rats were pretrained for staircase and rotarod tests. Behavior tests were performed eight times (i.e., pre-QA injection, post-TP 0, 2, 4, 6, 8, 10, and 12 weeks). Injections under general anesthesia were administered three times: QA injection for modeling (for all animals, *n* = 28), for TP of 1231A3-NPCs (*n* = 10), fibroblasts (*n* = 9), and media (*n* = 9), and for FG injection (*n* = 2 from each group).

FIGURE S2 Effects of 1231A3-NPCs on glial scar formation. **(A)** IHC staining with astroglial marker (GFAP). **(B)** Bar graph showing the rate of GFAP-positive cells (%). Scale bar = 50 μm, ^∗∗^*p* < 0.001.
