# Supplementary figures and images for: Neural Transplants From Human Induced Pluripotent Stem Cells Rescue the Pathology and Behavioral Defects in a Rodent Model of Huntington’s Disease
Source: Front Neurosci. 2020 Sep 18;14:558204. doi: 10.3389/fnins.2020.558204 (PMC7530284; doi:10.3389/fnins.2020.558204)

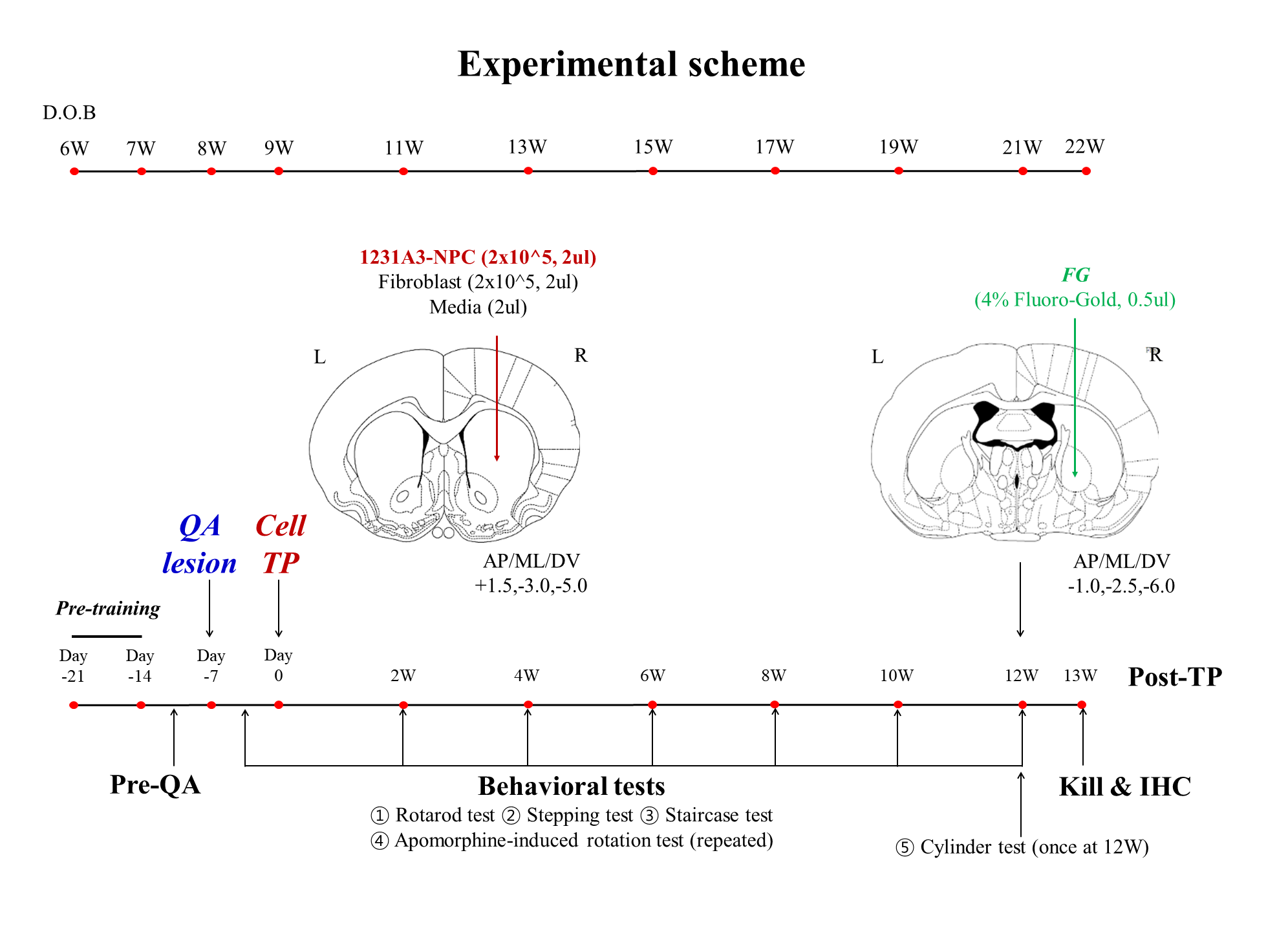

Supplement: FIGURE S1 — Schematic representation of experimental designs. Rats were pretrained for staircase and rotarod tests. Behavior tests were performed eight times (i.e., pre-QA injection, post-TP 0, 2, 4, 6, 8, 10, and 12 weeks). Injections under general anesthesia were administered three times: QA injection for modeling (for all animals, n = 28), for TP of 1231A3-NPCs (n = 10), fibroblasts (n = 9), and media (n = 9), and for FG injection (n = 2 from each group). [file Image_1.TIF]

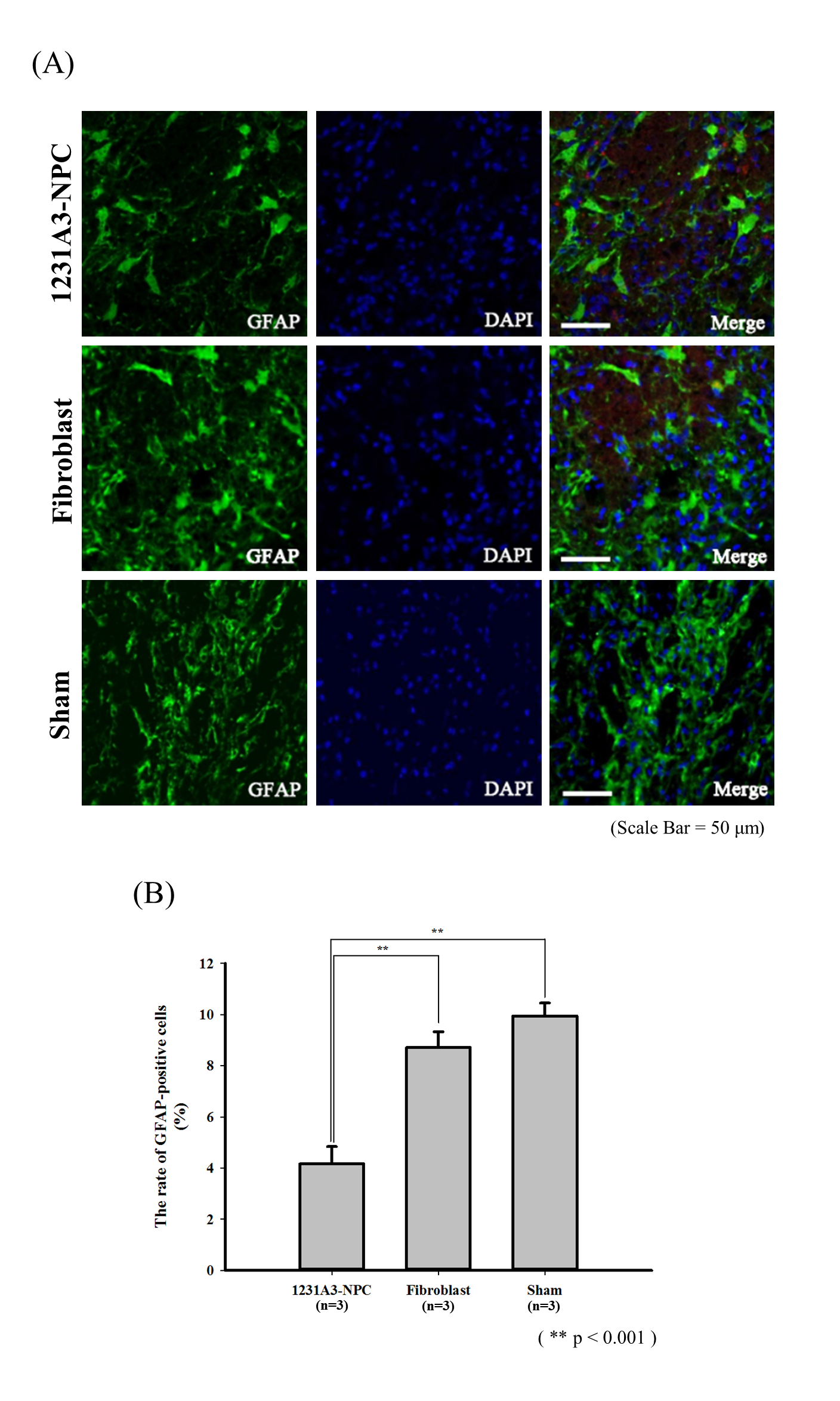

Supplement: FIGURE S2 — Effects of 1231A3-NPCs on glial scar formation. (A) IHC staining with astroglial marker (GFAP). (B) Bar graph showing the rate of GFAP-positive cells (%). Scale bar = 50 μm, ∗∗p < 0.001. [file Image_2.TIF]
